# Ultra-broadband and high-responsive photodetectors based on bismuth film at room temperature

**DOI:** 10.1038/srep12320

**Published:** 2015-07-21

**Authors:** J. D. Yao, J. M. Shao, G. W. Yang

**Affiliations:** 1 State Key Laboratory of Optoelectronic Materials and Technologies, Nanotechnology Research Center, School of Physics & Engineering, Sun Yat-sen University, Guangzhou 510275, Guangdong, P. R. China

## Abstract

Bismuth (Bi) has undergone researches for dozens of years on account of its abundant physics including the remarkably high mobility, exceptional large positive magnetoresistance and the coexistence of an insulating interior as well as metallic surfaces. Very recently, two-dimensional topologically-protected surface states immune to nonmagnetic perturbation such as surface oxidation and impurity scattering were experimentally demonstrated through systematic magnetotransport measurements, e.g. weak antilocalization effect and angular dependent Shubnikov-de Haas oscillations. Such robust metallic surface states, which are efficient in carrier transportation, along with its small bulk gap (14 meV) make Bi favored for high-responsive broadband photodetection. Here, we for the first time demonstrate the stable ultra-broadband photoresponse from 370 nm to 1550 nm with good reproducibility at room temperature based on a Bi photodetector. The fabricated device’s responsivity approaches 250 mA/W, accompanied with a rise time of 0.9 s and a decay time of 1.9 s. The photocurrent is linear dependent on the voltage and incident power, offering good tunability for multi-purpose applications. Thickness-dependent conductance and photocurrent reveal that the bulk is the optically active layer while the surface channel is responsible for carrier transportation. These findings pave an avenue to develop ultra-broadband Bi photodetectors for the next-generation multifunctional optoelectronic devices.

Bismuth (Bi) has undergone researches for dozens of years on account of its abundance physics. Despite substantial efforts, many fundamental properties of Bi still remain debated, e.g. an insulating interior but highly conductive surfaces, exceptional large positive magnetoresistance even at room temperature, and mobility far surpassing that of traditional materials^1^,^2^,^3^,^4^,^5^. Two dimensional (2D) materials such as transition-metal dichalcogenide (TMDs) and graphene have carved out significant inroad into photodetection due to their unique attributes, including large surface to volume ratio, flexibility as well as exceptional electrical and optical properties^6^,^7^,^8^,^9^,^10^,^11^. Though impressive progresses have been achieved, the sizable band gaps (i.e. ~1.8 eV for monolayer MoS_2_ and ~6 eV for monolayer BN) curtail their capacity of broadband photodetection^12^,^13^,^14^, which is at the heart of the multi-purpose technological applications such as imaging, communication and military surveillance^15^,^16^,^17^. Graphene is believed to be of great potential in broadband photodetection due to its unique gapless electronic structure^18^. However, its atomic thickness induced limited absorption (~2.3%) along with the ultrafast recombination of photogenerated carriers results in the suffering of poor responsivity (~mA/W)^16^,^19^,^20^, which can’t guarantee most practical needs. Thus, various approaches such as the integration of a waveguide, hybridization of quantum dots, band engineering and plasmonic have been exploited to improve the device performance. However, either the improvement is limited or the onerous processes add the complexity and degrade the practicality in device implementation^21^,^22^,^23^,^24^. Moreover, mass production, an essential requirement for device fabrication, appears to be unattainable due to the uncontrollable and nonrepeatability of the scotch-tape based micromechanical cleavage method, the most popular way to prepare 2D materials^13^,^14^. Therefore, the demand to explore new materials which are favorable for both broadband high performance photodetection and practicality in manufacture is becoming increasingly eminent.

Recently, systematic magnetotransport measurements, e.g. weak antilocalization effect (WAL) and angular dependent Shubnikov-de Haas (SdH) oscillations, have demonstrated Bi to be a topological insulator (TI) with 2D surface states immune to nonmagnetic perturbation such as surface oxidation and impurity scattering, like the most common TI Bi_2_(Te, Se)_3_^6^,^7^,^8^,^25^,^26^. Such findings reasonably explain the above mentioned novel properties of Bi and result in the resurgence of extensive scientific and engineering interest. Considering that graphene and TI Bi_2_Se_3_ have been proven to be highly competent for efficient electrodes, the Bi film should also show great promise in carrier transportation^15^,^27^,^28^. Superior to graphene^16^,^19^,^20^, the Bi film possesses an optically active bulk layer, which helps the absorption of incident light and hinders the ultrafast recombination of photogenerated carriers. That is, a Bi film itself is born in nature a hybrid structure on a par with the graphene-MoS_2_ heterojunction^29^. In addition, its relatively small bulk band gap (14 meV) ensures the capacity of broadband photoresponse, which tackles the detecting limitations of other 2D-material-based photodetectors. However, most of the efforts about Bi so far focused on its magnetotransport^4^,^5^,^6^,^7^, and the in-death exploration of its photoresponse is scarce.

Here, we demonstrate the stable ultra-broadband photoresponse from 370 nm to 1550 nm with good reproducibility at room-temperature of a Bi film. Its responsivity is about 250 mA/W, along with a rise time of 0.9 s and a decay time of 1.9 s. In addictions, our measurements show that the photocurrent exhibits a linear dependence on both the voltage and the incident power, offering good tunability for multi-purpose applications. Meanwhile, thickness-dependent conductance and photocurrent indicate that the bulk of Bi is the optically actively layer while the surface channel is responsible for carrier transportation. Therefore, these results stress that Bi opens up opportunities for developing the next generation ultra-broadband high performance photodetectors.

## Results

**Figure 1:**
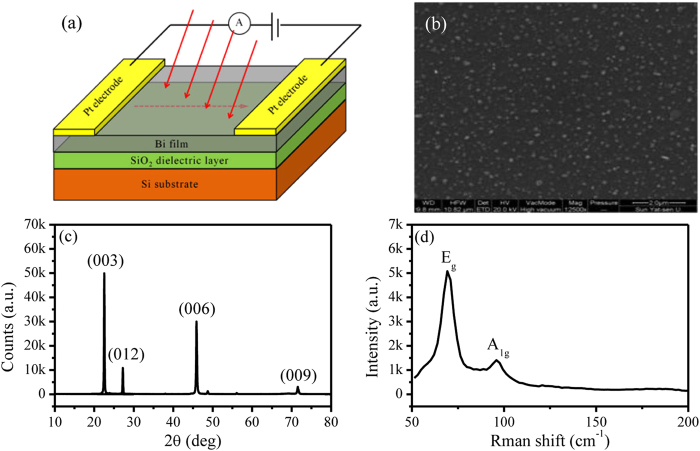
Schematic drawing of the Bi-based photodetector and characterization of the PLD-grown Bi film. (**a**) Three dimensional schematic view of the Bi photodetector. (**b**) SEM image, scale bar: 2 *μm*. (**c**) 2θ-ω X-ray diffraction pattern. (**d**) Raman spectroscopy with the 514 nm excitation laser.

The schematic diagram of a typical Bi photodetector is depicted in Fig. 1(a). The device is consisted of a PLD-grown Bi film and two Pt electrodes. Ohmic contact between the electrodes and the Bi film (Supplementary Information S1) is achieved to ensure to gain the intrinsic photoresponse from the Bi film instead of the heterojunction between the Bi film and the electrodes. The substrate used in the PLD-grown process is (100) oriented single crystal Si wafer with a 300 nm-thick SiO_2_ dielectric layer, which isolates the Bi film from the Si substrate to avoid the interference of the substrate channel on the transport measurements. Scanning electron microscope (SEM), Energy dispersive spectroscopy (EDS), Raman spectroscopy and X-ray diffraction (XRD) were conducted to study the morphology, constituent, structure and crystal quality of the PLD-grown Bi film. From a typical SEM image (Fig. 1b), it seems that the film possesses continuous surface morphology in large scale, with Bi grains randomly embedding in the surface. EDS analysis (Supplementary Information S2) suggests that the film only possess Bi atoms, indicative of its high purity. X-ray diffraction pattern (Fig. 1c) shows the pronounced (003) family diffraction peaks, suggesting that the sample crystallizes in the rhombohedral structure and is mostly c-axis oriented. To further confirm its structure, Raman scattering measurement with the 514 nm excitation laser is conducted (Fig. 1(d)). Obviously, there are two expected characteristic peaks at 69 cm^−1^ and 96 cm^−1^ , assigned as the 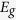 and 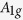
vibration mode of bismuth, respectively. Generally, all of the above-mentioned characterizations indicate that the PLD-grown Bi film is of high crystal quality and provides an attractive material platform to develop its potential opportunities.

**Figure 2:**
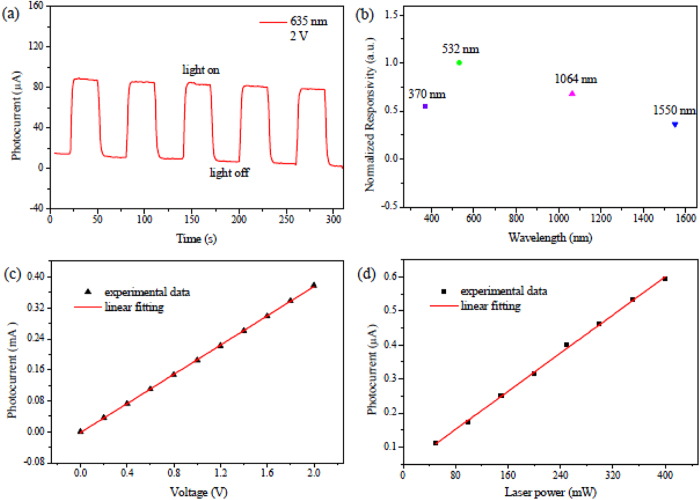
Photoresponse of the Bi photodetector. (**a**) Time dependent switching behavior of the photocurrent. Device area: 0.32 mm × 0.32 mm Power density: 300 mW/cm^2^. (**b**) Normalized responsivity as a function of illumination wavelength. Device size: 2 mm × 1.2 mm (**c**) Voltage dependent photocurrent. Device area : 10 mm × 10 mm (**d**) Power dependent photocurrent. Source-drain voltage: 0.01 V. Device area: 10 mm × 10 mm

Figure 2 summarizes the photoresponse of the fabricated Bi photodetector. As is shown Fig. 2(a), the device yields significant photocurrent under periodic illumination. The switching behavior, that is, the photocurrent ramps to a high level under illumination and annihilates after the remove of the illumination, is clearly observed. Unlike the reported photodetectors based on other 2D materials, in which there is obvious persistence photocurrent^12^,^14^, the photocurrent in the Bi photodetector saturates and decays quiet promptly. This is an indicative of little metastable charge trapping centers or local potential fluctuations caused by the material inhomogeneity, benefit from the high quality nature of the PLD-grown Bi film. The dynamic response of the device (Supplementary Information S3) reveals that the rise and decay times are 0.9 s and 1.9 s, respectively. Such a prompt speed is supposed to be facilitated by the efficient carrier transportation due to the Bi surface’s robustness and high mobility. Besides, during the multiple illumination cycles as shown in Fig. 2(a), the photocurrent responds in a similar fashion to the incident light with no significant variation on the signal amplitude. The responsivity extracted from it is c.a. 250 mA/W, far surpassing the initially reported devices based on other 2D materials, i.e. 1 mA/W for graphene and 7 mA/W for MoS_2_^13^,^16^. Such high responsivity is attributed to the combination of strong light-matter interaction in the insulating bulk and efficient transportation of photogenerated carriers by the conducting surface channel, which will be described in detail later. The photosensitivity, defined as 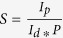, where I_p_, I_d_ , P, are photocurrent, dark current, incident light power density, respectively, is calculated to be 0.02 cm^2^/W, which seem to be quiet low compared to the traditional semiconducting material because of the little band gap of Bi. But it is bigger than that of graphene^30^. Besides, a Bi-based heterojunction can be exploited to improve the photosensitivity, like the pioneering works of graphene^31^.

From the spectral responsivity curve (Fig. 2(b)), we can see, in sharp contrast to those of other 2D materials, whose spectral responsivity exhibits a sharp cut-off at a relatively small wavelength due to their sizable gaps^32^,^33^, the Bi photodetector exhibits comparable response to incident light over an ultra-broadband range, also with good reproducibility (Supplementary Information S4), from ultraviolet (370 nm) to infrared (1550 nm). The responsivity reaches the maximum value at 532 nm and decrease gently away from it. This can be explained by the tradeoff between the kinetic energy and the number of the photogenerated carriers. For photons with higher energy, that is, shorter wavelength, more energy will be transferred to the photogenerated carriers. Therefore, they enjoy a larger probability to overcome the barrier blocking or defect/impurity trapping and finally contribute to the photocurrent. However, an increase of the photon energy is at the cost of its number under a constant incident power, which compromises the photocurrent’s increase. Therefore, the photocurrent is expected to reach a maximum value at a certain wavelength where the tradeoff is optimized.

To gain a deeper insight into the Bi photodetector, we further explored the influences of the other parameters including the driving voltage and the intensity of the incident light. Fig. 2(c) depicts the voltage dependent photocurrent. The photocurrent shows a linear dependence on the voltage. This is because larger external voltage blocks the recombination of photogenerated carriers more efficiently, thus resulting in easier carrier separation. Figure 2(d) shows the power dependent photocurrent. It is positively correlated with the laser power because a larger number of electron-hole pairs generates under a stronger illumination. The good linear output between the photocurrent and the illumination intensity again indicate that there are little trap states in the Bi photodetector.

## Discussion

**Figure 3:**
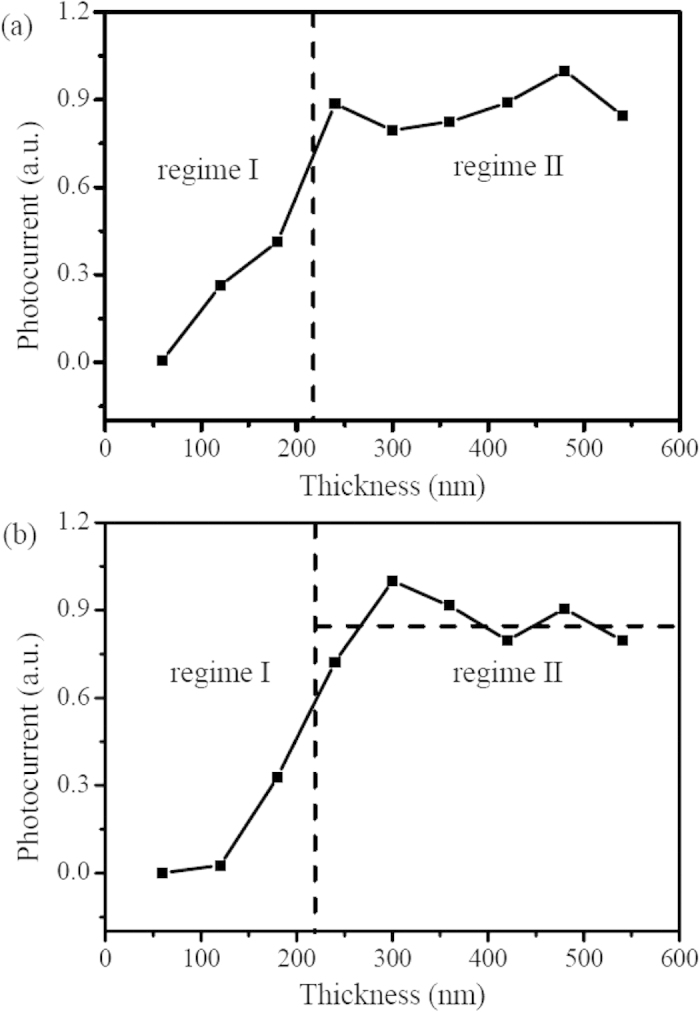
Dependence of the film’s performance on thickness. (**a**) Thickness dependent conductance. (**b**) Thickness dependent photocurrent. The size of the films is 2 mm × 1.2 mm Source-drain bias: 0.5 V.

To uncover the inherent physical operating mechanism of the Bi photodetector, its thickness-dependent conductance and photocurrent were investigated. As shown in Fig. 3(a), in general, the change in the conductance can be divided into two regimes, namely as the regime I and the regime II. In the regime I, i.e. the range thinner than 240 nm, the conductance increases promptly as the film’s thickness increases. This is due to the fact that better crystallinity can be achieved in a thicker film within such thickness range, thus leading to higher carrier mobility. (Supplementary Information S5).

In the regime II, the range thicker than 240 nm, an interesting result is presented. In stark contrast to the trivial materials, whose conductance is linear dependent on its thickness on account of a broader pass way^34^, the conductance of the Bi film appears to exhibit little thickness dependence. It is conceivable because Bi has been demonstrated to be TI with both metallic surface and insulating bulk, for which the traditional one channel model is not sufficient to describe it^2^,^6^,^7^. Therefore, a two channel model is proposed to elucidate such phenomenon (as depicted in detail in Supplementary Information S6). According to the model, the total conductance of the film can be expressed as 
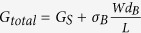
, 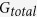 and 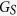 are the total and surface conductance, 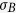 is the conductivity of the bulk; d_B_, W, L are the bulk thickness, width and length. For the traditional 3D TI Bi_2_(Se, Te)_3_, vacancies and antisite defects easily form^35^,^36^. These defects dope the film and shift the Fermi level from the insulating bulk gap to the conductance or valence band, which leads to the submergence of the surface states, i.e., the bulk channel dominants the total transportation. Therefore, the similar phenomenon has not been observed so far. However, the Bi film is born to by pass such predicament because it is consist of only one kind of atomic. As a result, its Fermi level lies in the gap and thus the surface channel is much conductive compared to the bulk and dominates the total transportation, resulting in 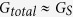. Consequently, the Bi film’s thickness-dependent conductance curve is relatively flat.

**Figure 4:**
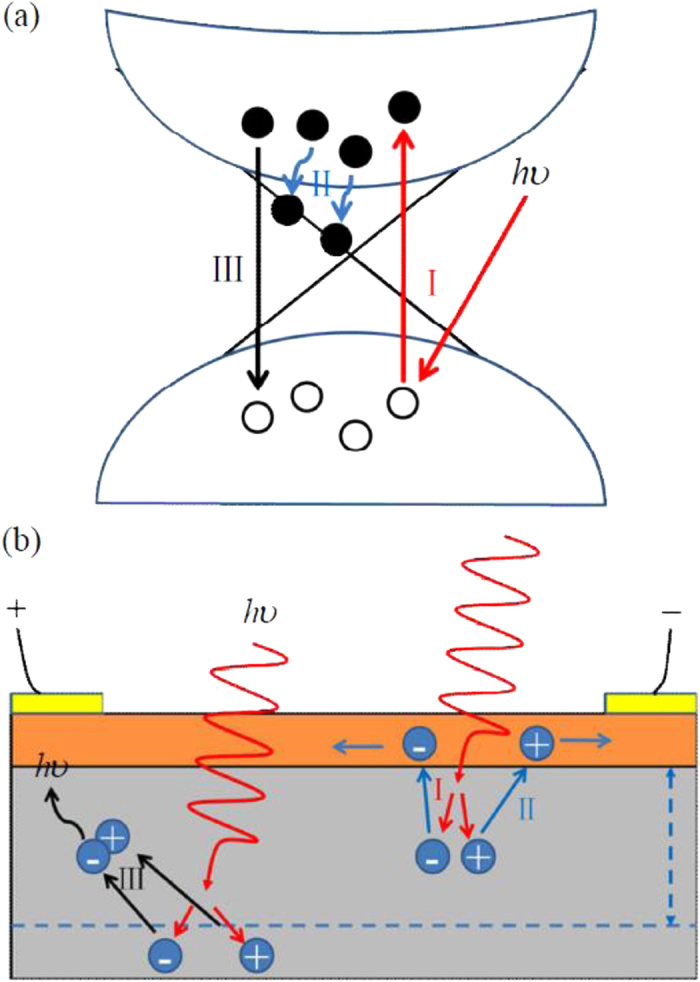
Operating mechanism of the Bi photodetector. Illustrations are shown in (**a**) reciprocal space and (**b**) real space, respectively. The steps indicated by I, II and III in (**a**) and (**b**) are corresponding steps in the two space.

Figure 3(b) shows the thickness-dependent photocurrent. It exhibits a prompt increase at the initial followed by a relatively flat tail. This is quiet a novel phenomenon because the films with larger thickness should absorb more photons and generate more electron-hole pairs, thus leading to larger photocurrent instead of a flat one^37^. Excitingly, this can also be well explained by the two channel nature of the Bi film, as schematically depicted in Fig. 4. Actually, the Bi film is composed of two functional portions, an insulating bulk and a metallic surface. The bulk is the broadband optically active layer due to the existence of a small band gap, while the surface is responsible for carrier collection and transportation because of its highly conductive properties originated from its embedded topologically nontrivial nature. On light illumination, electrons are excited from the valence band to the conductance band and electron-hole pairs generate, as depicted in the step I of Fig. 4(a,b). For the film thinner than one mean free path (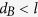), electron-hole pairs drift to the highly conductive surface channel as depicted in the step II of Fig. 4(a,b), which has been observed at TI Bi_2_Se_3_ by angle-resolved photoelectron spectroscopy (ARPES)^38^. Then, they are quickly transported to the electrodes by the external electric field, contributing to the photocurrent. Given the topologically nontrivial and highly conductive nature of the Bi film’s surface channel, the carrier transportation is efficient and therefore decent responsivity outperforming that of traditional 2D-material-based photodetectors is achieved. As the thickness of the film increases, more photons are absorbed and the number of the effective photogenerated carriers (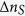) increases, resulting in a larger photocurrent according to 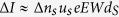 (Supplementary information S6).

For the film thicker than one mean free path (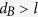), the operating mechanism of that portion within one mean free path remain the same. However, for the portion more than one mean free path away from the surface channel, electron-hole pairs usually recombine before they reach the surface conducting channel (the step III in Fig. 4(a,b)). Consequently, such portion contributes few photogenerated carriers to the total photoresponse of the Bi film, i.e.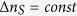, which means that photocurrent should not increase in this thickness range. Therefore, the measured photocurrent exhibits a saturated trend, as depicted in the regime II of Fig. 3(b).

In summary, we have fabricated a photodetector based on the PLD-grown Bi film and investigated its photoresponse. By harnessing the highly conductive nature of the surface and strong light-matter interaction of the bulk, the device achieves the stable ultra-broadband photoresponse from 370 nm to 1550 nm with responsivity approaching 250 mA/W as well as fast response/decay times of 0.9 s/ 1.9 s. The generality of these results suggest that high performance ultra-broadband Bi-based photodetector at room-temperature can be expected in the near future.

## Methods

### Materials preparation

PLD, a versatile deposition technology of high efficiency and repeatability, was exploited to prepare the high-quality Bi films in this work. The grown parameters are described as follows. The base pressure of the grown chamber is better than 1 × 10^−4^ Pa. The target material is consisted of highly pure and uniform Bi (99.99%). Prior to loading into the grown chamber, the substrates are cleaned in acetone followed by piranha solution (H_2_SO_4_ (98%): H_2_O_2_ (35%) = 3:1) in ultrasonic environment for 30 mins to remove the organic and inorganic contaminations, respectively. Finally, they are rinsed in deionized water to wash away the residue pollutant. Then, high-quality Bi films are deposited onto substrates at room temperature by PLD, and the working pressure is set at 30 Pa with flowing Ar_2_ at the rate of 50 sccm as the working gas.

### Measurements

A prototype photodetector is constructed by depositing Pt electrodes onto the surface of the Bi film with a shadow mask. The electrical characteristic of the Bi photodetector is evaluated using a Keithley 4200-SCS semiconductor parameter analyzer. All measurements are conducted at room-temperature under ambient condition.

## Additional Information

**How to cite this article**: Yao, J. D. *et al.* Ultra-broadband and high-responsive photodetectors based on bismuth film at room temperature. *Sci. Rep.* **5**, 12320; doi: 10.1038/srep12320 (2015).

## Supplementary Material

Supplementary Information
