# Supplementary material for: Ultra-broadband and high-responsive photodetectors based on bismuth film at room temperature
Source: Sci Rep. 2015 Jul 21;5:12320. doi: 10.1038/srep12320 (PMC4508833; doi:10.1038/srep12320)
Supplement: Supplementary Information [file srep12320-s1.doc]

**Supplementary Information for**

**Ultra-broadband and high-responsive photodetectors based on the bismuth film at room temperature**

J. D. Yao, J. M. Shao, G. W. Yang

**S1. Ohmic contact between the electrodes and the Bi film**

The current-voltage (I-V) characteristics of the Bi photodetector are shown in Fig. S1. The good linearity of the curve reveals good ohmic contact between the electrodes and the Bi film.

Figure S1. The current-voltage (I-V) characteristics of the Bi photodetector.

**S2. EDS analysis of the PLD-grown Bi film**

EDS analysis suggests that the film only possess Bi atoms, indicative of its high purity.

**
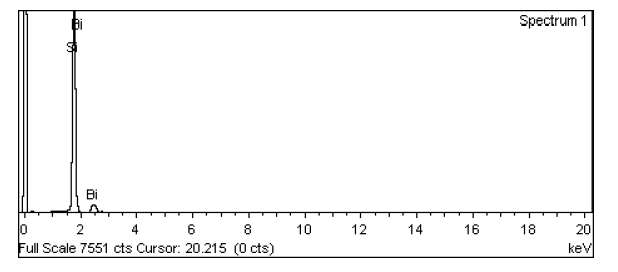
**

**
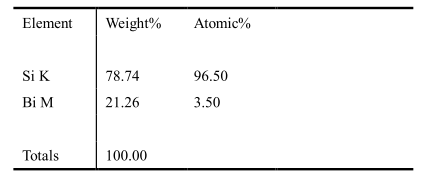
**

Figure S2. EDS analysis of the PLD-grown Bi film.

**S3. Dynamic response of the Bi photodetector**

The temporal photoresponse is depicted in Fig. S3. The time dependent photoresponse can be described by the following equations.

For the rise,

$I(t)=I_{0}+A_{0}[\exp(t/\tau_{rise})]$,

and for the decay,

$I(t)=I_{0}+A_{0}[\exp(-t/\tau_{decay})]$,

Where $\tau_{rise}$ is the response time, $\tau_{decay}$ is the recovery time, I_0_ is the dark current, and A_0_ is a constant. The response time and recovery time is calculated to be 0.9 s and 1.9 s, respectively.

(a)

(b)

(c)

**Figure S3.** The rising and falling times of the Bi photodetector. (a) A temporal photoresponse. The enlarged (b) rising and (c) falling edge of the data in (a). Source-drain bias: 0.05 V. Device size: $2\text{ mm }\times\text{ 1.2 mm}$.

**S4. Switching behavior of the Bi photodetector under illumination with wavelength from ultraviolet to infrared.**

Fig. S4 plots the switching behavior of the Bi photodetector under periodic illuminations. The result exhibits an excellent long-term reproducibility for all wavelengths, from ultraviolet to near-infrared, indicating its great potential for broadband photodetection.

Figure S4. Switching behavior of the Bi photodetector under illumination with wavelength of (a) 370 nm, (b) 532 nm, (c) 1064 nm, (d) 1550 nm. Source-drain bias: 0.05 V. Device area: $2\text{ mm }\times\text{ 1.2 mm.}$

**S5. XRD diffraction patterns of the Bi films with different thickness**

(b)

(c)

(a)

Figure S5. XRD diffraction peaks of the Bi films with the thickness of (a) 60 nm, (b) 120 nm, (c) 180 nm.

**S6. Two channel model for the Bi TI film**

The schematic diagram of the two channel model is illustrated in Fig. S4. The total conductance of the film is given by

$G_{total}=G_{S}+G_{B}=G_{S}+\sigma_{B}\frac{Wd_{B}}{L}$,

$G_{total}\text{, }G_{B}$ and $G_{S}$ are the total, bulk and surface conductance, $\sigma_{B}$ is the conductivity of the bulk; d_B_, W, L are the thickness, width and length, depicted in detail in Fig. S4. Considering that the surface channel is metallic while the bulk is insulating, i.e.$G_{S}>>G_{B}$, the total conductance can be express as:

$G_{total}\approx G_{S}$.

$G_{S}$ has no relationship with the film thickness, therefore the total conductance show little relationship with the film thickness.

Under certain bias U, the current can be expressed as

$I=I_{S}+I_{B}\approx I_{S}=\sigma_{S}ES=\frac{n_{S}e\mu_{S}UWd_{S}}{L}$.

On light illumination,

$I^{P}\approx\frac{n_{S}^{p}e\mu_{S}UWd_{S}}{L}$,

The photocurrent

$\Delta I\approx I^{P}-I=\frac{\Delta n_{S}e\mu_{S}UWd_{S}}{L}\text{, }\Delta\text{n}_{\text{S}}\text{=}n_{S}^{P}-n_{S}$.

Here, I_S_ and I_B_ are the surface and bulk current, E is the Electric field intensity along the channel,$\sigma_{S},\text{ }\mu_{S}\text{ and d}_{\text{S}}$ are the surface conductivity, mobility and thickness, respectively. $\text{n}_{\text{S}}\text{ and }n_{S}^{P}$ are the surface carrier density in dark and under illumination. Therefore, the photocurrent is positively correlated with the number of effective photogenerated carriers.

Figure S4. The schematic diagram of the two channel model for the TI Bi film.

Reference

1. S. Xiao, D. Wei, X. Jin, Bi(111) Thin Film with Insulating Interior but Metallic Surfaces. *Phys Rev Lett* **109**, 166805 (2012).
